# A novel cardiac magnetic resonance imaging technique to evaluate left ventricular diastolic function in patients with hypertrophic cardiomyopathy

**DOI:** 10.1186/1532-429X-14-S1-P162

**Published:** 2012-02-01

**Authors:** Shahryar G Saba, Sohae Chung, Robert Donnino, Monvadi B Srichai, Stuart Katz, Leon Axel

**Affiliations:** 1 New York University School of Medicine, New York, NY, USA

## Background

The temporal and spatial resolution of cardiac magnetic resonance (CMR) imaging allows precise tracking of the left ventricle (LV) atrioventricular junction (AVJ) throughout the cardiac cycle. Similar to tissue Doppler imaging in echocardiography, AVJ motion assessed by CMR reflects diastolic LV function. A prior study by our group demonstrated significant differences in the displacement and velocity of the AVJ during diastole in patients with heart failure with preserved ejection fraction compared to normal hearts. In the current study we hypothesized that diastolic left ventricular function defined by AVJ motion is abnormal in patients with hypertrophic cardiomyopathy (HCM), a group known to have altered LV relaxation properties.

## Methods

We retrospectively identified 24 patients with known or suspected HCM and normal ejection fractions who underwent CMR as part of their clinical assessment and 14 normal control subjects. The longitudinal motion of the lateral and septal AVJs was tracked at 25 times through the cardiac cycle using the 4-chamber cine CMR view . The baseline position of the AVJ was defined at end diastole and its longitudinal displacement was measured relative to a reference line drawn between the LV apex and the midpoint of the mitral annnulus. The displacement of the AVJ in relation to its starting position at end diastole was normalized by LV length. Based on the resulting plots of AVJ position versus time in the cardiac cycle (see figure below) five motion variables were calculated: maximum longitudinal displacement (MD) of the AVJ, maximum velocity during early diastole (MVED), velocity at half-maximal displacement during early diastole (VHMDED), slope of the best fit line of displacement in diastasis (VDS), and the ratio of VDS/MVED. Data obtained in HCM patients were compared to normal control subjects using the Student t-test.

**Figure 1:**
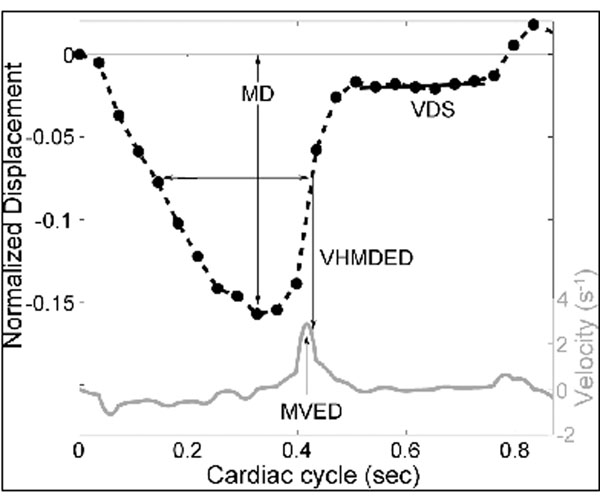
AVJ Position Versus Time

## Results

Means and standard deviations were calculated for each of the AVJ motion variables (MD, VHMDED, MVED, VDS, VDS/MVED) for the septal (S) and lateral walls (LW) of HCM patients and normal control subjects. Lateral and septal AVJ data were directly compared to the corresponding walls in patients and controls. We found highly statistically significant differences in MD and the four CMR correlates of diastolic LV function (VHMDED, MVED, VDS, VDS/MVED) in patients with HCM compared to normal controls, as noted in the table below.

**Table 1:** AVJ Motion Variables in HCM Patients and Control Subjects

	Control LW	HCM LW	*P*	Control S	HCM S	*P*
MD	-0.149 ± 0.029	-0.105 ± 0.027	<0.001	-0.154 ± 0.028	-0.112 ± 0.020	<0.001

VHMDED (s^-1^)	1.021 ± 0.316	0.464 ± 0.302	<0.001	0.772 ± 0.350	0.406 ± 0.216	<0.005

MVED (s^-1^)	1.083 ± 0.282	0.550 ± 0.266	<0.001	0.889 ± 0.291	0.521 ± 0.181	<0.001

VDS (s^-1^)	0.017 ± 0.026	0.097 ± 0.114	<0.005	0.036 ± 0.037	0.093 ± 0.081	<0.01

VDS/MVED	0.015 ± 0.026	0.195 ± 0.219	<0.001	0.035 ± 0.042	0.192 ± 0.175	<0.001

## Conclusions

Novel CMR correlates of diastolic left ventricular function are markedly abnormal in patients with HCM. Measured at the AVJ, patients with HCM had significantly less maximal displacements, slower velocities during early diastolic filling, and higher velocities during diastasis compared to normal control subjects. The evaluation of AVJ motion by CMR may provide a new method to assess diastolic function.

## Funding

NIH 1R21HL108218-01.

